# Tomographic Description of a Quantum Wave Packet in an Accelerated Frame

**DOI:** 10.3390/e23050636

**Published:** 2021-05-19

**Authors:** Sergio De Nicola, Renato Fedele, Dušan Jovanović, Margarita A. Man’ko, Vladimir I. Man’ko

**Affiliations:** 1 Dipartimento di Fisica “E. Pancini”, Universitá di Napoli Federico II, Complesso Universitario di M.S. Angelo, 80126 Napoli, Italy; renato.fedele@na.infn.it; 2 CNR-SPIN Unitá di Napoli, Complesso Universitario di M.S. Angelo, 80126 Napoli, Italy; 3 INFN Sezione di Napoli, Complesso Universitario di M.S. Angelo, 80126 Napoli, Italy; 4 Institute of Physics, University of Belgrade, 11080 Belgrade, Serbia; dusan.jovanovic@ipb.ac.rs; 5 P.N. Lebedev Physical Institute, 119991 Moscow, Russia; mankoma@lebedev.ru (M.A.M.); mankovi@lebedev.ru (V.I.M.)

**Keywords:** Radon transform, marginal distribution, equivalence principle, Schrödinger equation, accelerated frame

## Abstract

The tomography of a single quantum particle (i.e., a quantum wave packet) in an accelerated frame is studied. We write the Schrödinger equation in a moving reference frame in which acceleration is uniform in space and an arbitrary function of time. Then, we reduce such a problem to the study of spatiotemporal evolution of the wave packet in an inertial frame in the presence of a homogeneous force field but with an arbitrary time dependence. We demonstrate the existence of a Gaussian wave packet solution, for which the position and momentum uncertainties are unaffected by the uniform force field. This implies that, similar to in the case of a force-free motion, the uncertainty product is unaffected by acceleration. In addition, according to the Ehrenfest theorem, the wave packet centroid moves according to classic Newton’s law of a particle experiencing the effects of uniform acceleration. Furthermore, as in free motion, the wave packet exhibits a diffraction spread in the configuration space but not in momentum space. Then, using Radon transform, we determine the quantum tomogram of the Gaussian state evolution in the accelerated frame. Finally, we characterize the wave packet evolution in the accelerated frame in terms of optical and simplectic tomogram evolution in the related tomographic space.

## 1. Introduction

It is well known that quantum tomography provides a very useful probability representation of quantum states in terms of the so-called *marginal distribution* [1,2,3]. It has all of the features of a classical probability density [4], although it contains all information carried by a Wigner quasidistribution [5]. Thus, it is worth noting that, while the marginal distribution is positive definite, the Wigner quasidistribution is not positive definite. Marginal distribution and Wigner quasidistribution are related by one-to-one transformation, usually referred to as the Radon transform [6]. It has been proven [4] that the probability distribution for the rotated quadrature phase can be expressed in terms of the Glauber–Sudarshan distribution [7,8] (i.e., the *P*-function), the Husimi function [9] (*Q*-function), and the Wigner quasidistribution. Remarkably, the inverse of this statement is true as well, provided that the probability distribution for the rotated quadrature phase is known for each angle of rotation φ in the interval [0≤φ≤π] [4].

However, since there exists also an invertible transformation between Wigner quasidistribution and the wave function, the quantum states given in the configuration space can be directly characterized in terms of the tomographic representation, namely they are characterized by the tomographic probability density, usually referred to as the *tomogram* [10]. This approach received a great deal of attention in the literature [11], especially for its diverse applications in a number of quantum problems, ranging from linear to nonlinear quantum mechanics (see, e.g., [12,13] ).

Similar approaches have been also developed in the quantum-like domain to provide the tomographic probability representation of electromagnetic (EM) radiation. It is well known that the paraxial propagations of an EM beam in a linear or nonlinear medium is governed by suitable Schrödinger-like equations [14,15] for a wave function in which the squared modulus is proportional to the EM energy density (photon density). A Fresnel entropic characterization of optical Laguerre–Gaussian beams has been provided [16].

According to the Thermal Wave Model [17,18,19,20,21], a similar paraxial representation, based on a Schrödinger-like equation, can be adopted for relativistic charged particle (CP) beams while propagating in a vacuum or in other media. Therefore, the notion of a tomogram can be provided for the CP beams [22,23].

More recently, the concept of tomographic entropy has been proposed within the context of quantum tomography to characterize quantum states and related tomograms from the viewpoint of quantum information. Remarkably, the entropic uncertainty relations associated with a quantum state and the related tomogram have been also found and discussed [24,25].

Relevant mathematical and physical aspects of the tomograms and of the related entropy associated with the quantum states [1,2,3,4,5,6,7,8,9,10,11,12,13,14,15,16] are worth mentioning in the present context. (i) There exist symplectic tomograms and optical tomograms that are suitable for optics applications. (ii) The tomograms are related to the wave functions by invertible integral transforms associated with Radon transform and fractional Fourier transform for optical and symplectic tomograms. The paraxial evolution equations of the tomogram (marginal distribution) is governed by a Boltzmann-like equation in the space of tomographic parameters (i.e., tomographic space). It carries all information that is carried by the beam wave function as a solution to the (macroscopic) paraxial Schrödinger-like equation in the configuration space and by the Wigner quasidistribution as a solution to the Wigner–Moyal equation or the von Neumann-like equation in the phase space [26].

The aim of this paper is to carry out a theoretical investigation for the study, both analytical and numerical, of the spatiotemporal evolution of the tomogram associated with a single quantum particle (i.e., a quantum wave packet) in an accelerated frame in which the initial wave function is assumed to be Gaussian. In the following section, we present the formulation of our problem. Then, we reduce our problem to finding the spatiotemporal evolution of the quantum wave packet in an inertial frame in the presence of a uniform force field with an arbitrary time dependence of the acceleration. In Section 3, we show that such a wave packet remains Gaussian during the entire time evolution, i.e., the uncertainties of the position and of the momentum are unaffected by the uniform force field. Next, we describe the time evolution of the motion of both the envelope and the centroid. Similr to the case in free motion, the product of the position and momentum uncertainties is unaffected by the acceleration. Remarkably, the wave packet centroid is shown to move according to classic Newton’s law of motion of a single particle experiencing the effects of uniform acceleration, according to the Ehrenfest theorem. In Section 4, we extend our analysis to the tomographic space. We find the optical and the simplectic tomograms to be associated with the quantum wave packet. Some interesting examples for problems in the transport of charged and neutral particles are provided. Finally, remarks and conclusions are given in Section 5.

## 2. Formulation of the Problem

We want to study the 1D motion of a quantum particle of mass *m* that moves along the *z*-axis with nonrelativistic speed in an uniformly accelerated frame, where the latter accelerates along *z*. Let us denote by ψ(z,t) the wave function that characterizes the quantum wave packet associated with the particle, where its group velocity, i.e., the particle velocity, is given by $v=v\left(t\right)\;\hat{z}$. Here, *z* and *t* are space and time coordinates, respectively. In order to describe the spatiotemporal evolution of the quantum wave packet, we proceed according to the following steps: (i) we write the Schrödinger equation in the accelerated frame, which is specified above. (ii) We reduce our problem to solve the Schrödinger equation, as given in the inertial frame, for a quantum particle experiencing the following uniform force field $F\left(z,t\right)=F\left(t\right)\;\hat{z}$. Note that, correspondingly, the potential energy appearing in the transformed Schrödinger equation is of the form U(z,t)=−F(t)z.

### 2.1. The Schrödinger Equation for a Single Particle in an Accelerated Frame

According to the above notation, we write the Schrödinger equation for the single nonrelativistic particle describing free motion, i.e.,

(1) $$i\hbar \frac{\partial \psi }{\partial t}=-\frac{\hbar^{2}}{2m}\frac{\partial^{2}\psi }{\partial z^{2}},$$

that we cast as follows (with *m* being the single-particle rest mass):

(2) $$i\lambda_{c}\frac{\partial \Psi }{\partial s}=-\frac{\lambda_{c}^{2}}{2}\frac{\partial^{2}\Psi }{\partial z^{2}},$$

where we introduced the Compton wavelength, i.e., $\lambda_{c}=\hbar /mc$, and the time-like variable, i.e., s=ct (with *c* being the speed of light in vacuo) and set Ψ=Ψ(z,s)=ψ(z,s/c). In order to write Equation (2) in an accelerated frame, we introduce the following coordinate transformation:

(3) $$\begin{array}{ccc} z^{\prime } & = & z^{\prime }\left(z,s\right)=z+\zeta \left(s\right),\\ s^{\prime } & = & s^{\prime }\left(z,s\right)=s,\\ \Psi^{\prime }\left(z^{\prime },s^{\prime }\right) & = & \Psi \left(z\left(z^{\prime },s^{\prime }\right),s\left(z^{\prime },s^{\prime }\right)\right), \end{array}$$

where $z^{\prime }=z^{\prime }\left(z,s\right)$ and $s^{\prime }=s^{\prime }\left(z,s\right)$ denote the direct transformation whilst $z=z\left(z^{\prime },s^{\prime }\right)$ and $s=s\left(z^{\prime },s^{\prime }\right)$ denote the inverse one. Note that, if ζ(s) is a linear function of *s*, transformation (3) and its inverse reduces to Galilean transformations. Therefore, in order to write Equation (2) in an accelerated frame, we assume that at least $\ddot{\zeta }\neq 0$, where each dot stands for the first derivative with respect to the time-like variable *s*. By introducing Equation (3) into Equation (2) one easily arrives at

(4) $$i\lambda_{c}\left(\frac{\partial \Psi^{\prime }}{\partial s^{\prime }}+\dot{\zeta }\;\frac{\partial \Psi^{\prime }}{\partial z^{\prime }}\right)=-\frac{\lambda_{c}^{2}}{2}\frac{\partial^{2}\Psi^{\prime }}{\partial z^{\prime 2}}\;,$$

where the *dragging term*, i.e., $i\lambda_{c}\dot{\zeta }\;\partial \Psi^{\prime }/\partial z^{\prime }$, accounts for the velocity of the frame. Note that thearea accessible to the single-particle phase space is expressed as “length × angle”, namely “meters × radiants” (see, f.i., Schrödinger–Robertson uncertainty relation). Note that, if ζ(s) is a linear function of *s*, transformation (3) and its inverse reduces to Galilean transformations. However, this does not mean that the dragging term is zero.

### 2.2. Reduction to Motion in the Presence of a Uniform Force Field

In order to reduce Equation (4) to the Schrödinger equation for a single particle in the presence of a force field, we cast the wave function $\Psi^{\prime }\left(z^{\prime },s^{\prime }\right)$ in the following symplectic tomographic form:

(5) $$\Psi^{\prime }\left(z^{\prime },s^{\prime }\right)=\Phi \left(z^{\prime },s^{\prime }\right)\;\exp\left\{\frac{i}{\lambda_{c}}S\left(z^{\prime },s^{\prime }\right)\right\},$$

where $S\left(z^{\prime },s^{\prime }\right)$ is a real function. Then, by substituting Equation (5) into Equation (4), after some algebra, one finally obtains the following Schrödinger equation for $\Phi (x^{\prime },s^{\prime })$, viz.,

(6) $$i\lambda_{c}\frac{\partial \Phi }{\partial s^{\prime }}\;=\;-\frac{\lambda_{c}^{2}}{2}\frac{\partial^{2}\Phi }{\partial z^{\prime 2}}\;-\;\ddot{\zeta }\left(s^{\prime }\right)\;z^{\prime }\;\Phi$$

and an equation for $S\left(z^{\prime },s^{\prime }\right)$, viz.,

(7) $$S\left(z^{\prime },s^{\prime }\right)\;=\;-\dot{\zeta }\;z^{\prime }\;+\;\frac{1}{2}\;\int \;{\dot{\zeta }}^{2}\;ds^{\prime }\;.$$

Note that the last term on the right-hand side of Equation (6), i.e., $-\;\ddot{\zeta }\left(s\right)\;z^{\prime }$, can be thought of as the (dimensionless) potential energy term associated with the time-dependent uniform force. In other words, by means of transformation (5), the noninertiality of the system can be identified in such an energy potential term. Moreover, one can also think of Equation (6) as given in an inertial frame describing the spatiotemporal evolution of the wave function of a single particle in the presence of the force field $F\left(s\right)=\ddot{\zeta }\left(s\right)\;\hat{z}$ provided that the formal substitutions $z^{\prime }\to z$ and $s^{\prime }\to s$ are performed,

(8) $$i\lambda_{c}\frac{\partial \Phi }{\partial s}\;=\;-\frac{\lambda_{c}^{2}}{2}\frac{\partial^{2}\Phi }{\partial z^{2}}\;-\;\ddot{\zeta }\left(s\right)\;z\;\Phi \;.$$

Remarkably, according to the equivalence principle, one may also think of the above force term as the time-dependent uniform gravitational field generated by the acceleration of the frame or simply the uniform gravitational field generated by a suitable spatial mass distribution. Furthermore, if the single particle is also charged, one may identify the above force term as the one generated by an uniform electric field experienced by the quantum particle. The main property exhibited by Equation (5) is that ${\left|\Psi^{\prime }\left(z^{\prime },s^{\prime }\right)\right|}^{2}={\left|\Phi \left(z^{\prime },s^{\prime }\right)\right|}^{2}$. This implies the conservation of the probability density, namely, the conservation of the norm.

## 3. Gaussian Solution for an Arbitrary Acceleration

We look for a Gaussian wave packet solution to Equation (8), i.e.,

(9) $$\Phi \left(z,s\right)=\frac{1}{\sqrt[{4}]{2\pi \sigma^{2}\left(s\right)}}\;\exp\left\{\frac{i}{\lambda_{c}}\left[\alpha_{1}\left(s\right)z^{2}+\alpha_{2}\left(s\right)z+\alpha_{3}\left(s\right)\right]\right\}\;\exp\left\{\left[-\frac{{\left(z-\beta (s)\right)}^{2}}{4\sigma^{2}\left(s\right)}\right]\right\},$$

where σ(s), β(s), $\alpha_{1}\left(s\right)$, $\alpha_{2}\left(s\right)$, and $\alpha_{3}\left(s\right)$ are functions of a time-like variable to be determined. To this end, we substitute the expression of Φ on the right-hand side of Equation (8). Then, after some simple algebra, we arrive at the following set of constraints for the above functions:

(10) $$\begin{array}{ccc} \alpha_{1} & = & \frac{\dot{\sigma }}{2\;\sigma }\;, \end{array}$$

(11) $$\begin{array}{ccc} \alpha_{2} & = & \dot{\beta }-\beta \;\frac{\dot{\sigma }}{\sigma }\;, \end{array}$$

(12) $$\begin{array}{ccc} \dot{\alpha_{3}} & = & \frac{\lambda_{c}^{2}\;\beta^{2}-2\;\lambda_{c}^{2}\;\sigma^{2}-4\;{\alpha_{2}}^{2}\;\sigma^{4}}{8\sigma^{4}}\;, \end{array}$$

(13) $$\begin{array}{ccc} \ddot{\beta } & = & -\ddot{\zeta }\;, \end{array}$$

(14) $$\begin{array}{ccc} \ddot{\sigma } & = & \frac{\lambda_{c}^{2}}{4\;\sigma^{3}}\;. \end{array}$$

Equations (10)–(14) constitute a system of coupled ordinary differential equations to be satisfied for an arbitrary time dependence of the acceleration $\ddot{\zeta }=\ddot{\zeta }\left(s\right)$. Furthermore, note that Equation (14) is a particular case of the well-known Ermakov–Pinney equation [27,28] widely investigated in a number of problems concerning the time-dependent quantum harmonic oscillator and the search for operator invariants (see, f.i., [29])

Some remarks are in order.

(i). Φ(z,s) satisfies the normalization condition, i.e.,
  (15) $${\int \;|\Phi \left(z,s\right)|}^{2}\;dz=1\;.$$
  Therefore, Φ(z,s) represents the probability amplitude associated with the wave packet.
(ii). The average of the spatial coordinate *z* (i.e., 〈z〉, the wave packet centroid), with respect to Φ(z,s), defined as
  (16) $$\langle z\rangle ={\int \;z\;|\Phi \left(z,s\right)|}^{2}\;dz$$
  satisfies the property 〈z〉=β(s).
(iii). The length of the wave packet, i.e., the rms of the probability density, defined as
  (17) $$\sqrt{\langle {\left(z-\langle z\rangle \right)}^{2}\rangle }=\sqrt{\int \;{\left(\;z-\langle z\rangle \;\right)}^{2}\;{\left|\Phi \left(z,s\right)\right|}^{2}\;dz}\;,$$
  satisfies the property $\sqrt{\langle {\left(z-\langle z\rangle \right)}^{2}\rangle }=\sigma \left(s\right)$.
(iv). Note that Equation (14) can be easily integrated without assigning the explicit time dependence of $\ddot{\zeta }$. Furthermore, by multiplying Equation (14) by $\dot{\sigma }$, one finds the conserved quantity
  (18) $$E=\frac{1}{2}\;\dot{\sigma^{2}}\;+\;\frac{\lambda_{c}^{2}}{8\sigma^{2}}$$
  (i.e., energy conservation, $\dot{E}=0$). Then, by expanding the quantity $d^{2}\sigma^{2}/ds^{2}$ and by combining the result with Equation (14), we obtain
  (19) $$\frac{d^{2}\sigma^{2}}{ds^{2}}=4E\;,$$
  which can be immediately integrated providing
  (20) $$\sigma \left(s\right)=\sqrt{2Es^{2}+2\sigma_{0}\dot{\sigma_{0}}s+\sigma_{0}^{2}}\;,$$
  where we imposed the initial conditions, i.e., $\sigma_{0}=\sigma \left(s=0\right)$ and ${\dot{\sigma }}_{0}=\dot{\sigma }\left(s=0\right)$. It is worth mentioning that the solution for σ(s) does not depend on the force term appearing in Equation (8) and, therefore, it was integrated independently of the remaining differential equations of the system (10)–(14). This is due to the fact that the homogeneous force can act on the centroid of the wave packet but not on its rms.
(v). Moreover, we can formally integrate Equation (13) for β. Thus, imposing the initial conditions, i.e., $\beta_{0}=\beta \left(s=0\right)$ and $\dot{\beta_{0}}=\dot{\beta }\left(s=0\right)$, we obtain the solution
  (21) $$\beta \left(s\right)=-\int_{0}^{s}\;ds^{\prime }\;\int_{0}^{s^{\prime }}\;\ddot{\zeta }\left(s^{\prime \prime }\right)\;ds^{\prime \prime }\;\;+\;\dot{\beta_{0}}s\;+\;\beta_{0}\;.$$
  The double integration of $\ddot{\zeta }$ can readily give, viz.
  (22) $$\beta \left(s\right)=-\zeta \left(s\right)\;+\;\dot{\beta_{0}}s\;+\;\beta_{0}\;.$$
(vi). System of Equations (10)–(14) can be cast in such a way to express all the quantities $\alpha_{1}\left(s\right)$, $\alpha_{2}\left(s\right)$, and $\alpha_{3}\left(s\right)$ in terms of σ(s), $\dot{\sigma }\left(s\right)$, β(s), and $\dot{\beta }\left(s\right)$ only, i.e.,
  (23) $$\begin{array}{lll} \alpha_{1} & = & \frac{\dot{\sigma }}{2\;\sigma }\;, \end{array}$$
  (24) $$\begin{array}{lll} \alpha_{2} & = & \dot{\beta }-\beta \;\frac{\dot{\sigma }}{\sigma }\;, \end{array}$$
  (25) $$\begin{array}{lll} \alpha_{3} & = & \int_{0}^{s}\;{\left[\frac{\lambda_{c}^{2}\;\beta^{2}-2\;\lambda_{c}^{2}\;\sigma^{2}-4\sigma^{4}\;{\left(\dot{\beta }-\beta \;\dot{\sigma }/\sigma \right)}^{2}}{8\sigma^{4}}\right]}_{s=s^{\prime }}ds^{\prime }\;\;+\;\alpha_{30}\;\;, \end{array}$$
  where $\alpha_{30}\equiv \alpha_{3}\left(0\right)$. By substituting the solution σ(s) and the formal solutions β(s) in Equation (11), we obtain the formal solutions $\alpha_{1}\left(s\right)$, $\alpha_{2}\left(s\right)$, and $\alpha_{3}\left(s\right)$. In the next section, we find solutions for the latter in the case of constant acceleration.
(vii). The quadratic phase term $\alpha_{1}\left(s\right)\;z^{2}/\lambda_{c}$ appearing in Equation (9) defines a parabolic phase. It can be cast as $z^{2}/2\lambda_{c}\;\rho \left(s\right)$, where $1/\rho \left(s\right)=\dot{\sigma }\left(s\right)/\sigma \left(s\right)$ is the related bending radius; remarkably, it does not depend on the acceleration.
(viii). The linear phase term $\alpha_{2}\left(s\right)\;z/\lambda_{c}$ represents a plane phase contribution and depends on the acceleration.
(ix). The homogeneous phase term $\alpha_{3}\left(s\right)$ does not contribute to bending of the phase but depends on the acceleration.

### 3.1. Envelope Motion

In the previous section, we described the envelope motion. In particular, we found the envelope equation (Equation (19)) and its solution (Equation (20)) for an arbitrary acceleration. Since the energy constant of motion is positive, starting from the initial conditions $\sigma_{0}$ (which can be positive only), and ${\dot{\sigma }}_{0}=0$, in the configuration space, the wave packet can spread out only, similar to the absence of forces.

Let us denote the conjugate momentum of *z* by *p*, i.e., $p=\dot{z}$. Then, we introduce the momentum spread of the quantum wave packet, i.e.,

(26) $$\sigma_{p}^{2}\left(s\right)=\langle {\left(p-\langle p\rangle \right)}^{2}\rangle$$

and note that $\langle p\rangle =\langle \dot{z}\rangle =\dot{\beta }$. Therefore, Equation (26) can be expanded as

(27) $$\sigma_{p}^{2}\left(s\right)=\langle p^{2}\rangle -{\langle p\rangle }^{2}=\langle p^{2}\rangle -{\langle \dot{\beta }\rangle }^{2}\;,$$

where $\langle p^{2}\rangle$ can be given according to the quantum formalism of Equation (8) by

(28) $$\langle {\hat{p}}^{2}\rangle =\int_{-\infty }^{\infty }\Phi^{⋆}\left(-\lambda_{c}^{2}\frac{\partial^{2}}{\partial z^{2}}\right)\Phi \;dz=\lambda_{c}^{2}\int_{-\infty }^{\infty }{\left|\frac{\partial \Phi }{\partial z}\right|}^{2}\;dz\;.$$

Then, taking into account solution (9) and Equations (23) and (24), we easily obtain

(29) $$\langle {\hat{p}}^{2}\rangle ={\dot{\sigma }}^{2}+\frac{\lambda_{c}^{2}}{4\sigma^{2}}+{\dot{\beta }}^{2}\;,$$

which combined with Equation (51) provides

(30) $$\sigma_{p}^{2}\left(s\right)={\dot{\sigma }}^{2}+\frac{\lambda_{c}^{2}}{4\sigma^{2}}\;.$$

Consequently, by virtue of the conservation of energy, Equation (18) we finally obtain

(31) $$\sigma_{p}^{2}\left(s\right)=2\;E=const\;.$$

Therefore, starting from the initial conditions $\sigma \left(0\right)=\sigma_{0}$ and $\dot{\sigma }\left(0\right)={\dot{\sigma }}_{0}=0$ in the configuration space, the wave packet spreads out (i.e., σ is a function of time), whilst in the momentum space, it does not spread out (i.e., $\sigma_{p}$ does not change in time).

### 3.2. Motion of Centroid

According to the Ehrenfest theorem, the wave packet centroid moves with arbitrary acceleration and obeys the following Newton law for averaged quantities, according to Equation (13). Such a motion equation has to be solved with respect to β(s), according to Equation (22), once an arbitrary *1D* time-dependent acceleration, i.e., $-\ddot{\zeta }$, is assigned. This aspect suggests the possibility to investigate the wave packet evolution by controlling a specific acceleration of the centroid.

## 4. Gaussian Solution for Constant Acceleration

In this section, we find the Gaussian solution by assuming that the wave packet acceleration is constant, i.e.,

(32) $$\ddot{\zeta }=a=\mathrm{const}\;.$$

Consequently, we have

(33) $$\langle \;z\;\;\rangle \;=\;\beta \left(s\right)=-\frac{1}{2}a\;s^{2}\;+\;\dot{\beta_{0}}s\;+\;\beta_{0}\;.$$

According to Equations (23)–(25), the explicit solutions for $\alpha_{1}\left(s\right)$, $\alpha_{2}\left(s\right)$, and $\alpha_{3}\left(s\right)$, respectively, are straightforwardly determined, i.e.,

(34) $$\begin{array}{ccc} \alpha_{1}\left(s\right) & = & \frac{2Es+\sigma_{0}{\dot{\sigma }}_{0}}{2\left(2Es^{2}+2\sigma_{0}{\dot{\sigma }}_{0}s+\sigma_{0}^{2}\right)}, \end{array}$$

(35) $$\begin{array}{ccc} \alpha_{2}\left(s\right) & = & \left(-as+{\dot{\beta }}_{0}\right)+\left(\frac{1}{2}a\;s^{2}\;-\;\dot{\beta_{0}}s-\beta_{0}\right)\;\frac{2Es+\sigma_{0}{\dot{\sigma }}_{0}}{2Es^{2}+2\sigma_{0}{\dot{\sigma }}_{0}s+\sigma_{0}^{2}}\;, \end{array}$$

(36) $$\begin{array}{ccc} \alpha_{3}\left(s\right) & = & -\frac{a^{2}s^{3}}{24}-\frac{\beta_{0}as}{2}+\frac{\beta_{0}^{2}}{2s}-\frac{8a^{2}\lambda_{c}^{2}\sigma_{0}^{7}{\dot{\sigma }}_{0}}{{\left(\lambda_{c}^{2}+4\sigma_{0}^{2}{\dot{\sigma }}_{0}^{2}\right)}^{3}}+\frac{2a\sigma_{0}^{4}\left(-2{\dot{\beta }}_{0}\lambda_{c}^{2}+a\sigma_{0}^{3}{\dot{\sigma }}_{0}\right)}{{\left(\lambda_{c}^{2}+4\sigma_{0}^{2}{\dot{\sigma }}_{0}^{2}\right)}^{2}}\\ & + & \frac{2\sigma_{0}^{3}\left(a{\dot{\beta }}_{0}\sigma_{0}-a\beta_{0}{\dot{\sigma }}_{0}+{\dot{\beta }}_{0}^{2}{\dot{\sigma }}_{0}\right)}{\lambda_{c}^{2}+4\sigma_{0}^{2}{\dot{\sigma }}_{0}^{2}}-\frac{{\left[as^{2}-2\left(\beta_{0}+{\dot{\beta }}_{0}s\right)\right]}^{2}\sigma_{0}^{3}\left(\sigma_{0}+{\dot{\sigma }}_{0}s\right)}{2s\left[4\sigma_{0}^{4}+8\sigma_{0}^{3}{\dot{\sigma }}_{0}s+\left(\lambda_{c}^{2}+4\sigma_{0}^{2}{\dot{\sigma }}_{0}^{2}\right)s^{2}\right]}\\ & - & \frac{\lambda_{c}}{2}\arctan\left(\frac{\lambda_{c}^{2}s/\sigma_{0}^{2}+4\sigma_{0}{\dot{\sigma }}_{0}+4{\dot{\sigma }}_{0}^{2}s}{2\lambda_{c}}\right)\;. \end{array}$$

Note that

$$\begin{array}{ccc} \alpha_{30} & = & \lim_{s\to 0}\alpha_{3}\left(s\right)=-\beta_{0}{\dot{\beta }}_{0}+\frac{\beta_{0}^{2}{\dot{\sigma }}_{0}}{2\sigma_{0}}-\frac{8a^{2}\lambda_{c}^{2}\sigma_{0}^{7}{\dot{\sigma }}_{0}}{{\left(\lambda_{c}^{2}+4\sigma_{0}^{2}{\dot{\sigma }}_{0}^{2}\right)}^{3}}+\frac{2a\sigma_{0}^{4}\left(-2\lambda_{c}^{2}{\dot{\beta }}_{0}+a\sigma_{0}^{3}{\dot{\sigma }}_{0}\right)}{{\left(\lambda_{c}^{2}+4\sigma_{0}^{2}{\dot{\sigma }}_{0}^{2}\right)}^{2}}\\ & + & \frac{2\sigma_{0}^{3}\left(a{\dot{\beta }}_{0}\sigma_{0}-a\beta_{0}{\dot{\sigma }}_{0}+{\dot{\sigma }}_{0}^{2}\right)}{\lambda_{c}^{2}+4\sigma_{0}^{2}{\dot{\sigma }}_{0}^{2}}-\frac{\lambda_{c}}{2}\arctan\left(\frac{2\sigma_{0}{\dot{\sigma }}_{0}}{\lambda_{c}}\right)\;. \end{array}$$

## 5. Tomographic Representation of the Quantum Wave Packet

### 5.1. Density Matrix ρ (i.e., Density Operator $\hat{\rho }$) and Wigner Function *W*

It is well known from quantum mechanics that the Wigner function [5] can be defined as a sort of Fourier transform of the *density matrix* $\rho (z,z^{\prime },s)$, where *z* and $z^{\prime }$ are the coordinates of two points of the configuration space. Therefore, ρ can be also regarded as a two-point correlation function and its spatiotemporal evolution is governed by the following equation [30]

(37) $$i\lambda_{c}\frac{\partial \rho (z,z^{\prime },s)}{\partial s}=\left(\hat{H}-{\hat{H}}^{{}^{\prime }*}\right)\rho \left(z,z^{\prime },s\right)\;,$$

where $\hat{H}$ is an arbitrary Hamiltonian operator that has been made dimensionless by dividing by the rest energy (i.e., $mc^{2}$). Remarkably, ρ satisfies the following properties: (i) ρ(z,z,s))≥0; (ii) $\rho *\left(z,z^{\prime },s\right)=\rho \left(z^{\prime },z,s\right)$; (iii) for a pure state, that is associated with the wave function Ψ(z,s), $\rho \left(z,z^{\prime },s\right)=\Psi \left(z,s\right)\Psi^{*}\left(z^{\prime },s\right)$; (iv) ∫W(z,p,s)dzdp≡1. The Wigner function and its inverse are then defined respectively as follows:

(38) $$W\left(z,\;p,\;s\right)=\frac{1}{2\pi \lambda_{c}}\;\int \rho \left(z+\frac{u}{2},z-\frac{u}{2},s\right)\exp\left[-\frac{i}{\lambda_{c}}p\;u\right]\;du\;,$$

and

(39) $$\rho \left(z,z^{\prime },s\right)=\int W\left(\frac{z+z^{\prime }}{2},\;p,s\right)\exp\left[\frac{i}{\lambda_{c}}p\;\left(z^{\prime }-z\right)\right]\;dp\;.$$

### 5.2. Marginal Distribution $w\left(Z\right)$

For an arbitrary Hermitian operator $\hat{O}$, we can introduce the characteristic function, χ(k), given by $\chi \left(k\right)=\langle e^{ik\hat{O}}\rangle \equiv T_{r}\left(\hat{\rho }\;e^{ik\hat{O}}\right)$, where $T_{r}$ stands for the trace operator. Note that the inverse Fourier transform of χ(k), i.e.,

(40) $$w\left(Z\right)=\frac{1}{2\pi }\int \chi \left(k\right)e^{+ikZ}dk,$$

obeys to the following properties: (i) $w\left(Z\right)=\langle Z|\hat{\rho }|Z\rangle \geq 0$; (ii) ∫w(Z)dZ=1, where we took into account the properties of the diagonal element of $\hat{\rho }$.

In other words, w(Z) has the features of a classical probability density. In particular, if we define the characteristic function χ(k) to be the appropriate Fourier transform of the Wigner function, we can write Equation (40) as

(41) $$w\left(Z,µ,\nu \right)=\frac{1}{2\pi }\int W\left(z,p,s\right)\exp\left\{ik\left[Z-µ\;z-\lambda_{c}\;\nu p\right]\right\}\;dk\;dz\;dp\;.$$

In turn, Equation (41) can be cast as follows:

(42) $$w\left(Z,µ,\nu \right)=\int \;W\left(q,p\right)\delta \left(Z-µ\;q-\;\lambda_{c}\nu \;p\right)\;dq\;dp.$$

It is worth noting that this result shows that, according to the classical picture of tomogram, the quantum tomography given in the form containing the δ-function represents still a collection of projections and rotations based on the concept of probability density with all of the features of a classical distribution. Furthermore, the physical meaning of such a tomogram clearly shows that the above integration over dk is not trivial providing physical meaning of the quantum-state in terms of Wigner function.

Then, the physical meaning of function (41) is as follows. Let the parameters µ=cosθ and parameter ν=sinθ. This means that the parameter *Z* has the physical meaning of the position measured in the reference frame in the phase space with rotated initial axes of the position z and momentum p, i.e., Z=zcosθ+psinθ. We take $\lambda_{c}=1$ in (41) for simplicity. If the parameters µ and ν are arbitrary real numbers, we can write them as µ=Scosθ and $\nu =S^{-1}\sin\theta .$ This means that the parameter S is the scaling parameter of position and momentum. In this picture, the parameter *Z* is the position measured in the reference frame in the phase space, which was first rescaled (*z* became Sz and *p* became $S^{-1}p$ and then rotated by angle θ). It is important that the tomogram of Wigner function, which takes negative values, is also only a nonnegative probability distribution of the position measured in a scaled and rotated reference frame. Equation (41) defines the Radon transform, which is essentially a one-to-one mapping between the Wigner quasidistribution *W* and the marginal distribution *w*, in the literature usually referred to as the tomogram. For practical purposes, it is convenient to use the tomogram *w* for the description of the quantum wave packet in the space of the tomographic parameters µ,ν, that are hereafter referred to as the tomographic space or the T-space. Obviously, all information carried out by the Wigner function *W* are contained also in the tomogram *w*, but the latter possesses the advantageous features of a classical probability density. Most importantly, *w* is positive definite, which makes it accessible to direct measurements in a realistic laboratory experiment.

For an arbitrary potential energy U(z,s), the tomogram w(Z,µ,ν,s) and the quasidistribution W(z,p,s) are governed by transport equations that can be cast, respectively, as follows [1,2,3]:

(43) $$\left\{\frac{\partial }{\partial s}-\frac{µ}{\lambda_{c}}\frac{\partial }{\partial \nu }-\frac{i}{\lambda_{c}}\left[U\left({\left(\frac{\partial }{\partial Z}\right)}^{-1}\frac{\partial }{\partial µ}\;+\;i\frac{\lambda_{c}^{2}\nu }{2}\frac{\partial }{\partial Z},s\right)\;-\;U\left({\left(\frac{\partial }{\partial Z}\right)}^{-1}\frac{\partial }{\partial µ}\;-\;i\frac{\lambda_{c}^{2}\nu }{2}\frac{\partial }{\partial Z},s\right)\right]\right\}w=0$$

and

(44) $$\left\{\frac{\partial }{\partial s}+p\;\frac{\partial }{\partial z}+\frac{i}{\lambda_{c}}\left[U\left(z+\;i\frac{\lambda_{c}}{2}\frac{\partial }{\partial p},s\right)\;-\;U\left(z-i\frac{\lambda_{c}}{2}\frac{\partial }{\partial p},s\right)\right]\right\}W=0\;,$$

where ${\left(\partial /\partial Z\right)}^{-1}$ stands for the integral operator with respect to *Z*. Note that, since the tomogram (or the marginal distribution), *w* is the function of the variables (Z,µ,ν,s), viz. w=w(Z,µ.ν,s), the spatiotemporal tomographic evolution of the wave packet takes place in the (Z,s)-space for any fixed pair of tomographic parameters (µ,ν). Furthermore, since W=W(z,p,s), the spatiotemporal evolution of the Wigner quasidistribution takes place in the (z,p)-space at any time *s*.

For the case of homogeneous force field, i.e., $U\left(z,s\right)=-\ddot{\zeta }\left(s\right)\;z$ (see Section 2), Equations (43) and (44), respectively, take the particularly simple forms of the Boltzmann transport equations for the tomogram *w* in the tomographic space of variables (s,ν,Z), and for the Wigner function *W* in the phase space (s,z,p), respectively, viz.

(45) $$\left\{\frac{\partial }{\partial s}-\frac{µ}{\lambda_{c}}\frac{\partial }{\partial \nu }+\ddot{\beta }\left(s\right)\;\lambda_{c}\;\nu \frac{\partial }{\partial Z}\right\}\;w=0\;,$$

where Equation (13) was used, and

(46) $$\left\{\frac{\partial }{\partial s}\;+p\;\frac{\partial }{\partial z}-\ddot{\beta }\left(s\right)\;\frac{\partial }{\partial p}\right\}\;W=0\;.$$

It is easy to see that both Equations (45) and (46) possess, respectively, the following Gaussian and bi-Gaussian solutions that can be cast:

(47) $$w\left(Z,µ,\nu ,s\right)=\frac{1}{\sqrt{2\pi \sigma_{Z}^{2}\left(µ,\nu ,s\right)}}\;\exp\left\{-\frac{{\left(Z-\bar{Z}\right)}^{2}}{2\;\sigma_{Z}^{2}\left(µ,\nu ,s\right)}\right\},$$

(48) $$\begin{array}{ccc} & & W\left(z,p,s\right)=\\ & & \frac{1}{\pi \lambda_{c}}\exp\left\{-\frac{2}{\lambda_{c}^{2}}\left[\sigma_{p}^{2}\left(s\right){\left(z-\beta \left(s\right)\right)}^{2}-2\sigma_{zp}\left(s\right)\left(z-\beta \left(s\right)\right)\left(p-\dot{\beta }\left(s\right)\right)+\sigma_{z}^{2}\left(s\right){\left(p-\dot{\beta }\left(s\right)\right)}^{2}\right]\right\}\;, \end{array}$$

where $\bar{Z}=\langle Z\rangle =µ\langle z\rangle +\lambda_{c}\nu \langle p\rangle \;=\;µ\beta +\lambda_{c}\nu \langle \dot{\beta }\rangle$, $\bar{z}=\langle z\rangle =\beta \left(s\right)$, $\bar{p}=\langle p\rangle =\dot{\beta }\left(s\right)$, and

(49) $$\begin{array}{ccc} \sigma_{Z}\left(µ,\nu ,s\right) & = & \sqrt{\langle {\left(Z-\bar{Z}\right)}^{2}\rangle }=\sqrt{\frac{\lambda_{c}^{4}\nu^{2}}{4\sigma^{2}\left(s\right)}+{\left[µ\sigma \left(s\right)+\lambda_{c}\nu {\dot{\sigma }}^{2}\left(s\right)\right]}^{2}}\;, \end{array}$$

(50) $$\begin{array}{ccc} \sigma_{z}\left(s\right) & = & \sqrt{\langle {\left(z-\bar{z}\right)}^{2}\rangle }\;=\;\sigma \left(s\right)\;, \end{array}$$

(51) $$\begin{array}{ccc} \sigma_{p}\left(s\right) & = & \sqrt{\langle {\left(p-\bar{p}\right)}^{2}\rangle }\;=\;\sqrt{{\dot{\sigma }}^{2}\left(s\right)\;+\;\frac{\lambda_{c}^{2}}{4\sigma^{2}\left(s\right)}}\;, \end{array}$$

(52) $$\begin{array}{ccc} \sigma_{zp}\left(s\right) & = & \sqrt{\langle \left(z-\bar{z}\right)\;\left(p-\bar{p}\right)\rangle }=\sigma \left(s\right)\;\dot{\sigma }\left(s\right)\;. \end{array}$$

Note the following:

(i) By combining Equations (50)–(52), we easily obtain the well-known Robertson–Schrödinger uncertainty principle, i.e.,

$$\sigma_{z}^{2}\left(s\right)\sigma_{p}^{2}\left(s\right)-\sigma_{zp}^{2}\left(s\right)=\frac{\lambda_{c}^{2}}{4};$$

(ii) By means of a phase-space rotation, Equation (48) can be easily cast in a “canonical” form, in which the single Gaussian profiles in the configuration and momentum spaces do not entangle, i.e.,

$$\sigma_{zp}\;=\;0$$

(no correlation).

(iii) Equations (45) and (46) are sort of collisionless Boltzmann equations governing the spatiotemporal evolution of the marginal distribution w(Z,µ,ν,s) and the Wigner quasidistribution W(z,p,s), respectively. However, this analogy cannot be stressed much more because the role played by the canonical variables (z and p) in the Boltzmann equation differs from the one played by the tomographic parameters (µ and ν) in Equation (45). In fact, the canonical variables are related to each other through the Hamilton equations. The tomographic parameters, instead, are not, in principle, connected each other. Actually, each particular connection or, eventually, each set of conditions to be satisfied fixes the type of tomography; the related tomogram characterizes a particular tomographic description. In the literature, several types of tomography have been introduced; among them, one is very relevant for optical applications. It is called Fresnel tomography and can be obtained by assuming that ν is an arbitrary variable and µ satisfies the conditions µ=1. Alternatively, if we adopt µ=cosφ and ν=sinφ, we adopt the so-called optical tomography.

It is worth noting that there is a connection that relates the tomogram directly to the configuration space, namely, relates the wave function ψ to the tomogram *w*. Such a mapping can be rewritten in the following form [31]:

(53) $$w\left(Z,µ,\nu ,s\right)=\frac{1}{2\pi \lambda_{c}^{2}\left|\nu \right|}{\left|\int \psi \left(z,s\right)\exp\left[\frac{i}{2\lambda_{c}^{2}\nu }\left(µz^{2}-2Zz\right)\right]\;dz\;\right|}^{2}\;.$$

The *Fresnel tomogram* is obtained by imposing µ=1 in Equation (53), when it readily reduces to

(54) $$w\left(Z,1,\nu ,s\right)=\frac{1}{2\pi \lambda_{c}^{2}\left|\nu \right|}{\left|\int \psi \left(z,s\right)\exp\left[\frac{i}{2}\left(\frac{z^{2}}{\lambda_{c}^{2}\nu }-\frac{2Zz}{\lambda_{c}^{2}\nu }\right)\right]\;dz\;\right|}^{2}\;.$$

The evolution of the Fresnel tomographic map along the direction of propagation of the wave packet is determined by the variation in the field along the longitudinal coordinate *z*, i.e., by the evolution equation for the field.

Alternatively, by means of the transformation µ=µ(r,φ), ν=ν(r,φ) defined using

(55) µ=rcosφ,ν=rsinφ,

i.e., using cylindrical coordinates in the space of tomographic parameters (µ,ν), Equation (53) can be cast in a more suitable form, i.e.,

(56) $$w\left(Z,r,\varphi ,s\right)=\frac{1}{2\pi \lambda_{c}^{2}\left|r\sin\varphi \right|}{\left|\int \psi \left(z,s\right)\exp\left[\frac{i}{2\lambda_{c}^{2}}\left(\frac{z^{2}}{\tan\varphi }-\frac{2Zz}{\lambda_{c}^{2}r\sin\varphi }\right)\right]\;dz\;\right|}^{2}\;.$$

As we have discussed above, one of the tomographic parameters r,φ can be adopted arbitrarily. When we conveniently set r=1, we obtain the so-called *optical tomogram*, viz.,

(57) $$w\left(Z,1,\varphi ,s\right)=\frac{1}{2\pi \lambda_{c}^{2}\left|\sin\varphi \right|}{\left|\int \psi \left(z,s\right)\exp\left[\frac{i}{2\lambda_{c}^{2}}\left(\frac{z^{2}}{\tan\varphi }-\frac{2Zz}{\lambda_{c}^{2}\sin\varphi }\right)\right]\;dz\;\right|}^{2}\;.$$

## 6. Tomographic Characterization of the Wave Packet

In this section, we characterize the wave-packet spatiotemporal evolution in several cases of time-dependent acceleration. This is performed by providing a comparison among the probability density distributions that are described by the square modulus of the wave function ${\left|\Phi \left(z,s\right)\right|}^{2}$, the tomogram w(Z,µ,ν,s), and the Wigner quasiprobability density W(z,p,s). As discussed in the preceding section, these distributions provide exactly the same information about the physical system described by the quantum wave packet under study although they are defined in different spaces of independent variables that also have different physical meaning. Nevertheless, their diversity is shown to be useful to provide a better tomographic characterization of the quantum wave packet. In particular, here, we provide some numerical evaluations for the case of constant acceleration. Furthermore, in Section 7, we consider two interesting cases of non-constant acceleration: the case of a temporal burst of acceleration (Section 7.1) and the case of sinusoidal acceleration (Section 7.2).

### 6.1. Configuration Space Analysis

In the Madelung representation of the wave function, the solution Φ(z,s) of Equation (8) can be written in terms of the effective density ρ and the effective current velocity *V*, that are given in terms of the modulus and the phase of the wave function Φ as $\rho \left(z,s\right)\equiv {\left|\Phi \left(z,s\right)\right|}^{2}$ and V(z,s)≡∂φ(z,s)/∂z, respectively, where $\varphi \left(z,s\right)=\left(\lambda_{c}/i\right)\ln\left(\Phi /|\Phi |\right)$. Then, the effective density and the velocity of the Madelung fluid associated with the wave function of a quantum particle subjected to a time dependent, spatially uniform force, is readily obtained using Equations (9)–(14), viz.

(58) $$\begin{array}{ccc} \rho (z,s) & = & \frac{1}{\sqrt{2\pi \sigma^{2}\left(s\right)}}\;\exp\left\{\left[-\frac{{\left[z-\beta (s)\right]}^{2}}{2\sigma^{2}\left(s\right)}\right]\right\}, \end{array}$$

(59) $$\begin{array}{ccc} V(z,s) & = & \frac{\dot{\sigma }\left(s\right)}{\;\sigma (s)}\;\left[z-\beta (s)\right]+\dot{\beta }\left(s\right).\; \end{array}$$

**Figure 1:**
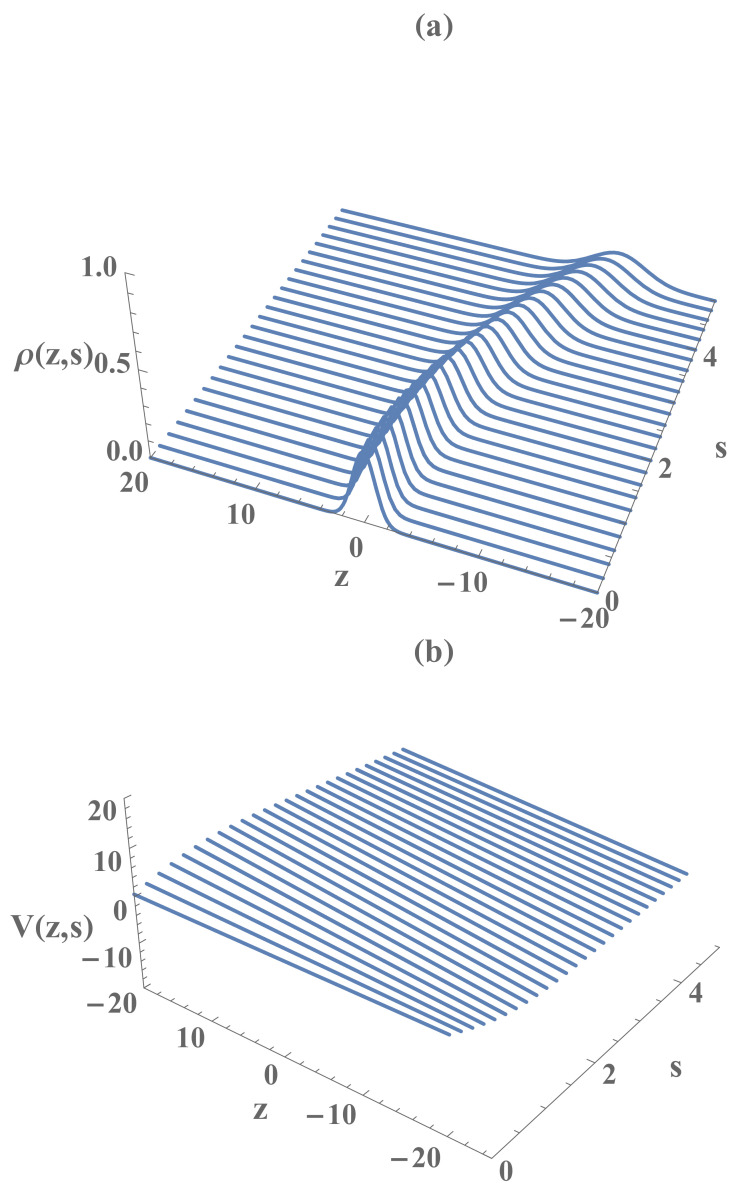
The Madelung fluid effective density ρ (**a**) and the effective current velocity *V* (**b**), as the functions of *z* and *s*, respectively, in the case of constant acceleration. The parameters are adopted as a=1.0, $\beta_{0}=0$, ${\dot{\beta }}_{0}=0.5$, $\sigma_{0}=1.0$, ${\dot{\sigma }}_{0}=0$, and $\lambda_{c}\equiv 1$.

**Figure 2:**
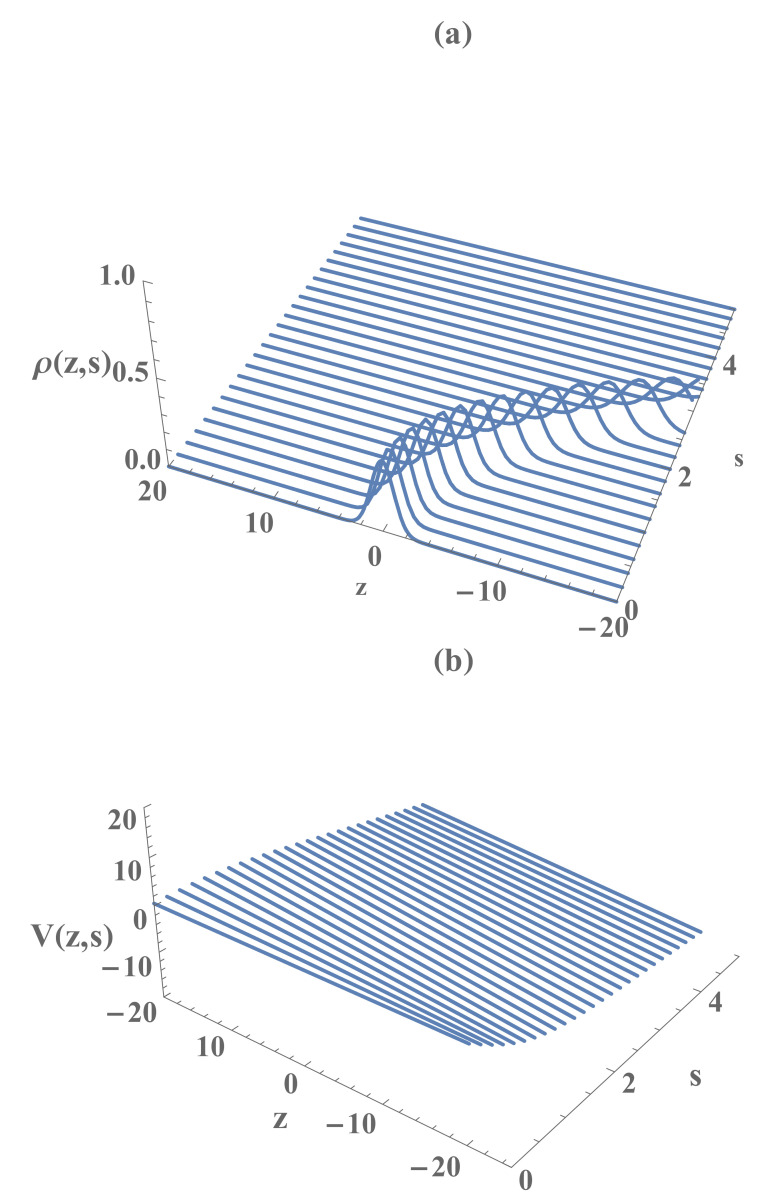
The Madelung fluid effective density ρ (**a**) and the effective current velocity *V* (**b**), as the functions of *z* and *s*, respectively, in the case of constant acceleration, where a=5.0, $\beta_{0}=0$, ${\dot{\beta }}_{0}=0.5$, $\sigma_{0}=1.0$, ${\dot{\sigma }}_{0}=0$, and $\lambda_{c}\equiv 1$.

Figure 1 and Figure 2 display ρ and *V* as functions of *z* and *s*, respectively, for the case of constant acceleration (i.e., $\ddot{\zeta }\;=\;const\equiv a$). From Equation (58), we readily find that the spatiotemporal variation of the probability density ρ(z,s) can be fully described by the temporal evolution of its profile (i.e., σ(s) ) located at its centroid (i.e., β(s)) that moves according to the classical equation of motion (22). Furthermore, according to Equation (59), the average of V(z,s) in the configuration space coincides with $\dot{\beta }\left(s\right)$, i.e., $\langle V(z,s)\rangle =\dot{\beta }\left(s\right)$. Therefore, it represents the velocity field in which the average corresponds to the most probable trajectory of the quantum particle in the configuration space (z,s). A simple quantum interpretation of the Madelung fluid asserts that, due to the quantum diffraction, the profiles ρ(z,s) and V(z,s) disperse around the most probable spatiotemporal trajectories or *spatiotemporal lines of current* of the corresponding quantum wave packet. Therefore, the envelope of such lines generate the spatiotemporal profiles of the quantum state (ρ,V) in the Madelung representation.

### 6.2. Phase Space Analysis

**Figure 3:**
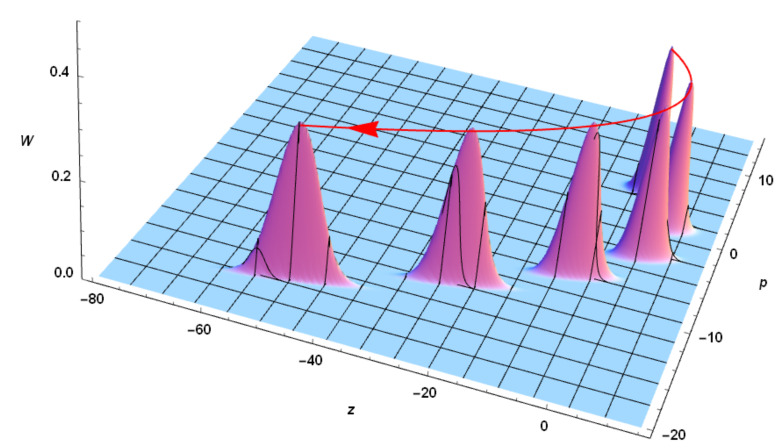
Wigner description of the spatiotemporal evolution of the quantum wave packet. Here, a=2, $\beta_{0}=0$, ${\dot{\beta }}_{0}=5.0$, $\sigma_{0}=0.5$, ${\dot{\sigma }}_{0}=0$, and $\lambda_{c}\equiv 1$. A sample of Wigner quasidistribution is displayed at different positions of the (z,p)-phase space. Note that, according to Ehrenfest theorem, the Wigner function centroid describes a phase-space parabola.

One can represent in the phase space the quantum state of the wave packet in terms of the Wigner quasidistribution given by Equation (48), which is the solution to Equation (46). The spatiotemporal evolution of the latter is illustrated by Figure 3 for a constant acceleration.

### 6.3. Tomographic Space

**Figure 4:**
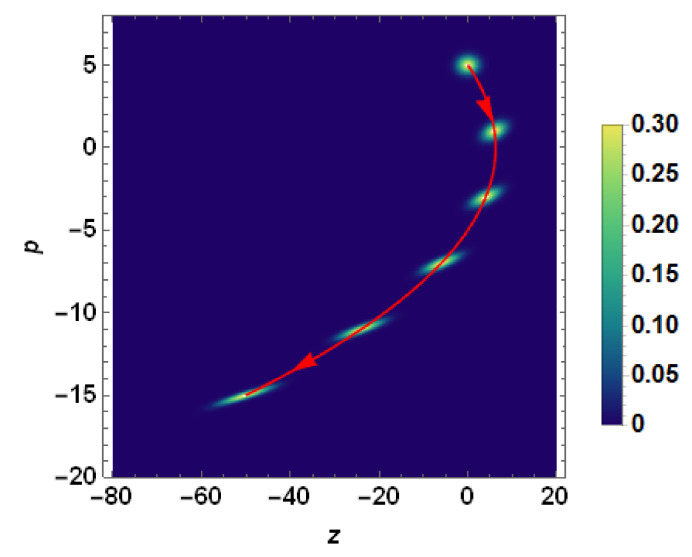
Wigner description of the spatiotemporal evolution of the quantum wave packet. Here, a=2, $\beta_{0}=0$, ${\dot{\beta }}_{0}=5.0$, $\sigma_{0}=0.5$, ${\dot{\sigma }}_{0}=0$, and $\lambda_{c}\equiv 1$. A sample of the phase-space density plot associated with the Wigner quasidistribution is displayed at different positions of the (z,p)-phase space. Note that, according to the Ehrenfest theorem, the wave packet centroid describes a phase-space parabola.

**Figure 5:**
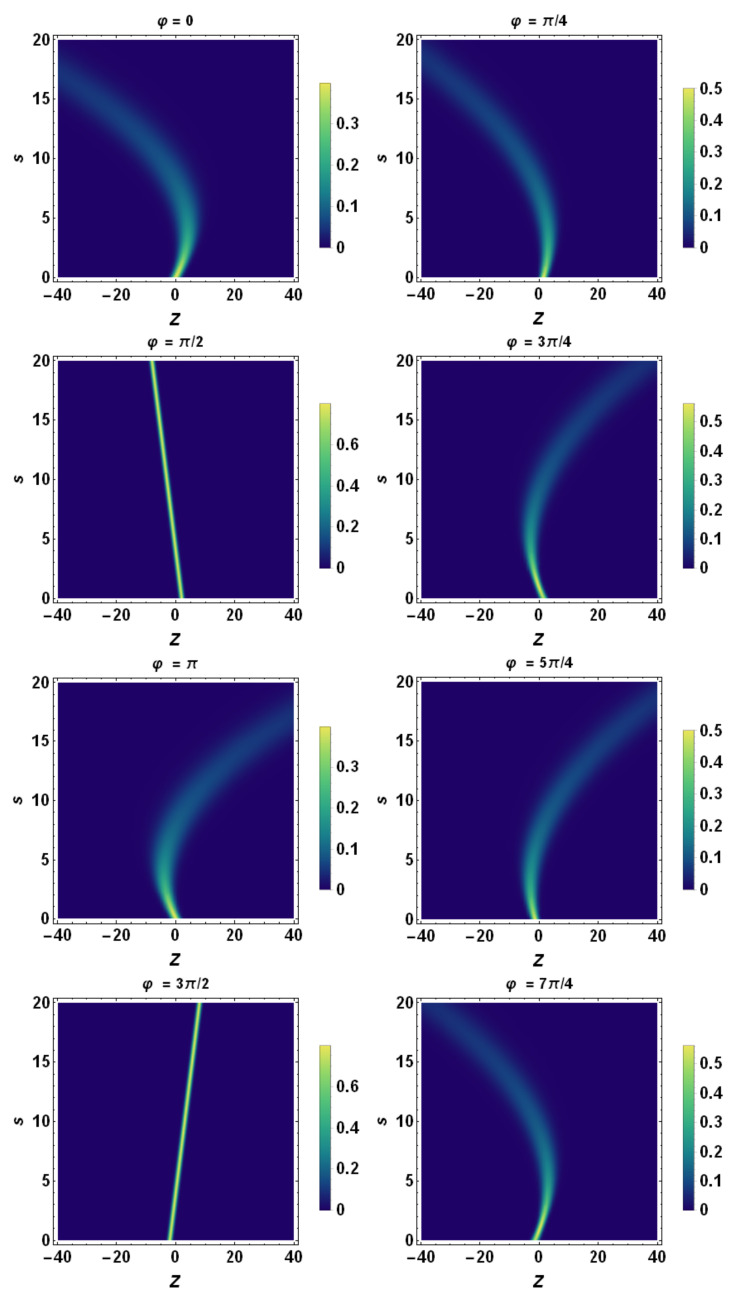
Density plot representation of tomogram as a function of *Z* and *s* for different angles of φ. Here, a=0.5, $\beta_{0}=0$, ${\dot{\beta }}_{0}=2.0$, $\sigma_{0}=1.0$, ${\dot{\sigma }}_{0}=0$, and $\lambda_{c}\equiv 1$. A sample of parabolas at different angles is displayed, except for φ=π/2 and φ=3π/2 when they reduce to straight lines. Each tomogram undergoes a progressive quantum spreading, except for φ=π/2 and φ=3π/2, when it remains fixed. According to Figure 7, the *Z*-axis coincides with $\lambda \dot{z}$ and the tomogram’s spreading reduces to the one of momentum space, i.e., of $\sigma_{p}$. Consequently, this quantity is conserved during the evolution of the wavepacket.

**Figure 6:**
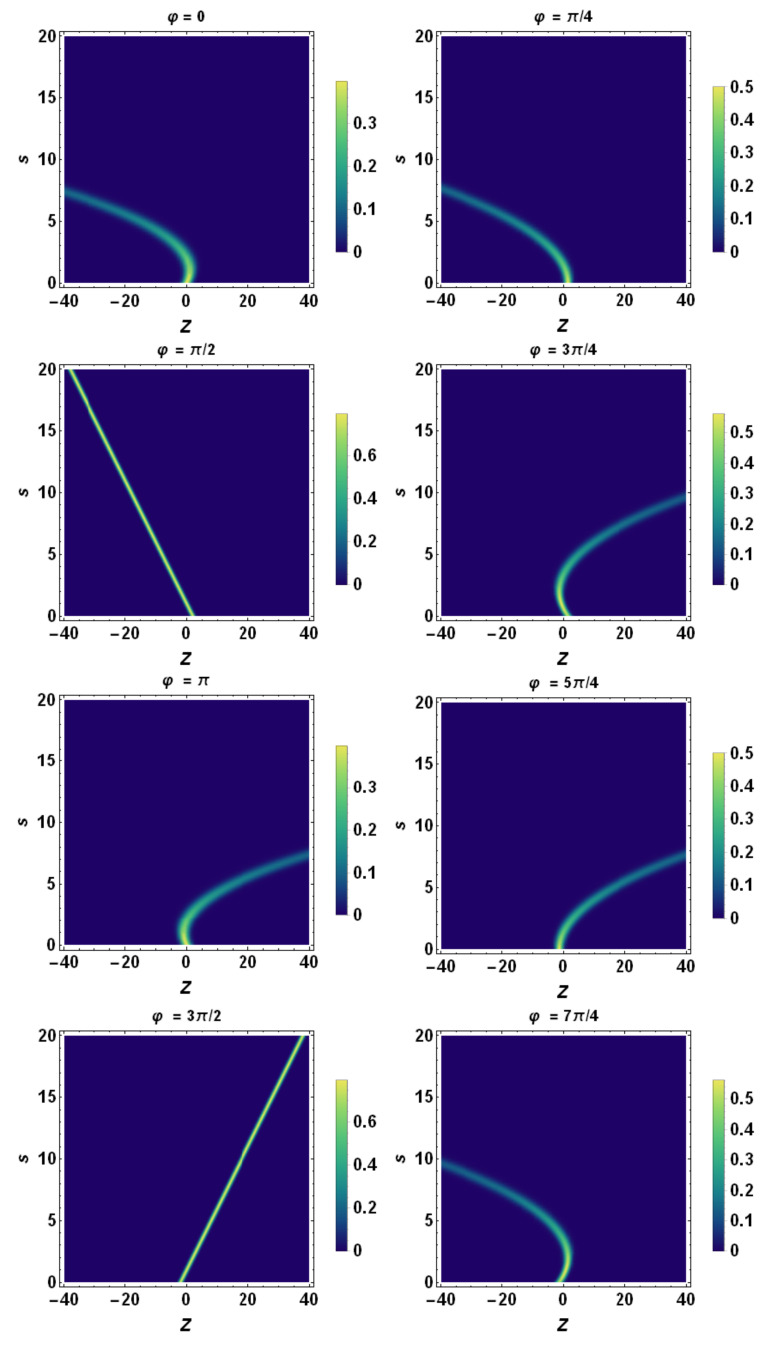
Density plot representation of tomogram as a function of *Z* and *s* for different angles φ. Here, a=2.0, $\beta_{0}=0$, ${\dot{\beta }}_{0}=2.0$, $\sigma_{0}=1.0$, ${\dot{\sigma }}_{0}=0$, and $\lambda_{c}\equiv 1$. A sample of parabolas at different angles is displayed, except for the cases φ=π/2 and φ=3π/2 when they reduce to straight lines. Each tomogram undergoes a progressive quantum spreading, except for φ=π/2 and φ=3π/2, where it remains fixed. According to Figure 7, the *Z*-axis coincides with $\lambda \dot{z}$ and the tomogram’s spreading reduces to the one of momentum space, i.e., of $\sigma_{p}$. Consequently, this quantity is conserved during the evolution of the wavepacket.

Finally, using the tomographic mapping given by Equation (53), we transform the spatiotemporal evolution of the quantum wave packet described in terms of the wave function (or, equivalently, in terms of the Wigner quasidistribution) into a suitable representation via its tomogram. As an example, our Figure 4, Figure 5 and Figure 6 display the tomographic spatiotemporal evolution of the quantum wave packet subjected to a constant acceleration, represented in the space of tomographic variables (Z,φ,s).

**Figure 7:**
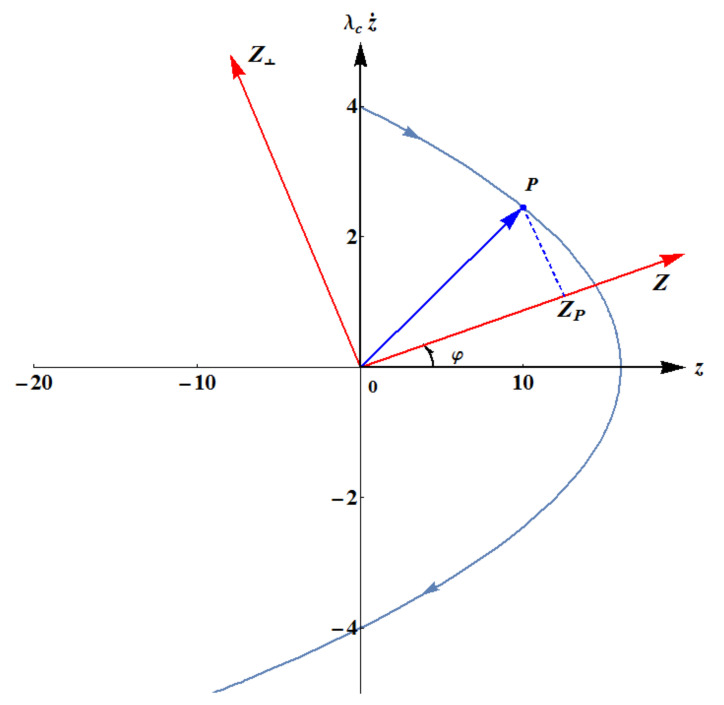
Geometric construction of the tomographic map.

The geometric construction of a symplectic tomogram for the case of constant acceleration is shown in Figure 7. The arrowed blue line is the trajectory of the centroid in the phase space ($z,\lambda_{c}\dot{z}$) calculated for constant a=0.5. Obviously, in the presence of a constant acceleration, the centroid of the wavepacket ($\langle z\rangle$, $\langle \lambda_{c}\dot{z}\rangle$) = ($\beta ,\lambda_{c}\dot{\beta }$) executes a parabolic motion. According to the Ehrenfest theorem, the latter represents the Newtonian aspect of such a motion, i.e., it coincides with the most probable trajectory of the quantum particle. Starting with the initial velocity ${\dot{z}}_{0}=4.0$, the centroid reaches the vertex of the parabola with a zero velocity and subsequently reverses its motion, propagating in the negative direction of the *z*-axis. The quantum aspect of such a process lies in the possibility that the particle might also follow (albeit, with smaller probabilities) all other possible orbits that are dispersed around the centroid trajectory.

**Figure 8:**
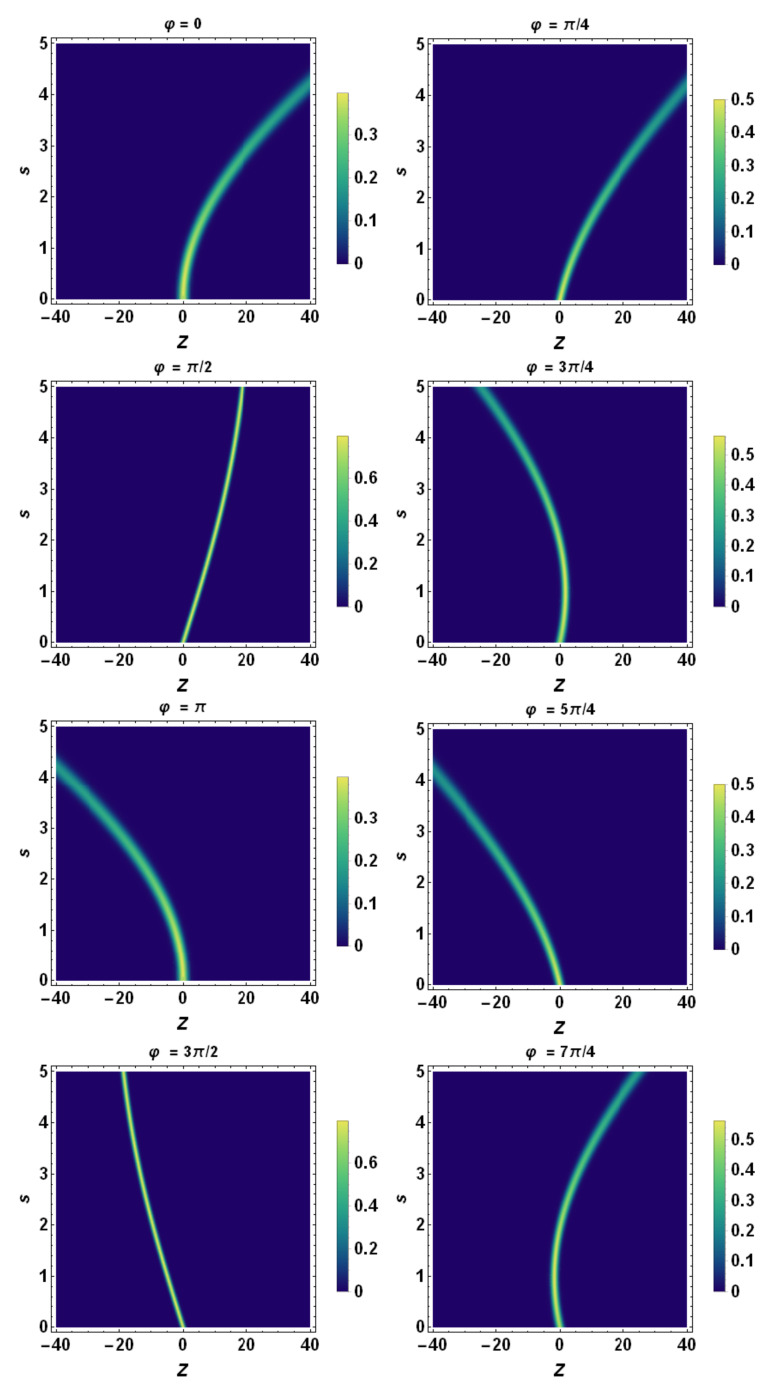
Density plot representation of tomogram as a function of *Z* and *s* for different angles φ. Here, we chose a temporal acceleration burst with Gaussian profile, i.e., $a\left(s\right)=a_{0}\exp\left(-s^{2}/\tau^{2}\right)$ (impulsive acceleration), with $a_{0}=5$ and τ=5. $\beta_{0}=0$, ${\dot{\beta }}_{0}=2.0$, $\sigma_{0}=1.0$, ${\dot{\sigma }}_{0}=0$, and $\lambda_{c}\equiv 1$. Note that the tomograms exhibit a bending due to a non-constant acceleration. As in the case of constant acceleration, the tomograms corresponding to φ=π/2 and φ=3π/2 exhibit fixed spreading whilst the ones corresponding to all the other values of φ spread out with a progressive increasing of the rms.

**Figure 9:**
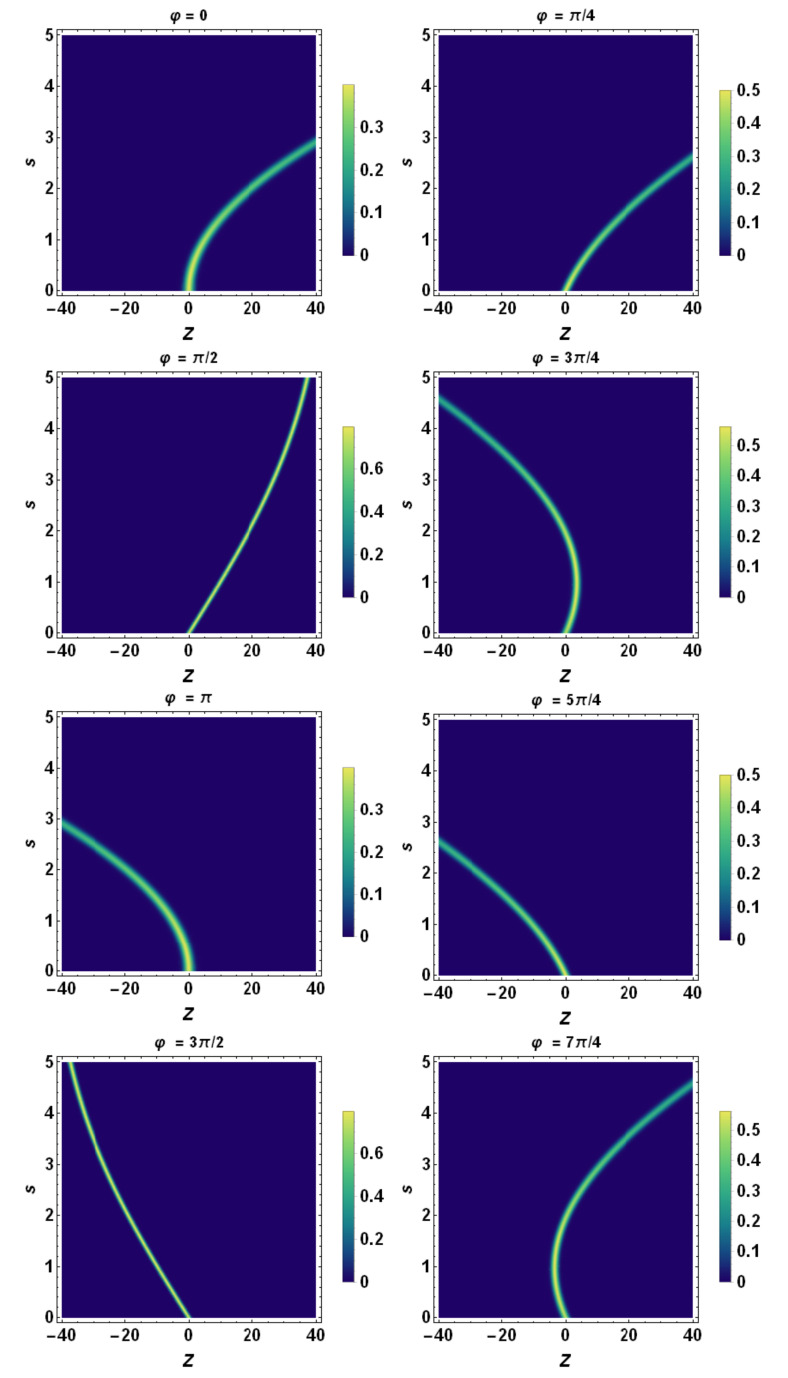
Density plot representation of tomogram as a function of *Z* and *s* for different angles φ. Here, we chose a non-constant acceleration with Gaussian profile, i.e., $a\left(s\right)=a_{0}\exp\left(-s^{2}/\tau^{2}\right)$, with $a_{0}=10$ and τ=5. $\beta_{0}=0$, ${\dot{\beta }}_{0}=2.0$, $\sigma_{0}=1.0$, ${\dot{\sigma }}_{0}=0$, and $\lambda_{c}\equiv 1$. Note that the tomograms exhibit bending due to non-constant acceleration. As in the case of constant acceleration, the tomograms corresponding to φ=π/2 and φ=3π/2 exhibit fixed spreading whilst the ones corresponding to all the other values of φ spread out with a progressive increasing of the rms.

**Figure 10:**
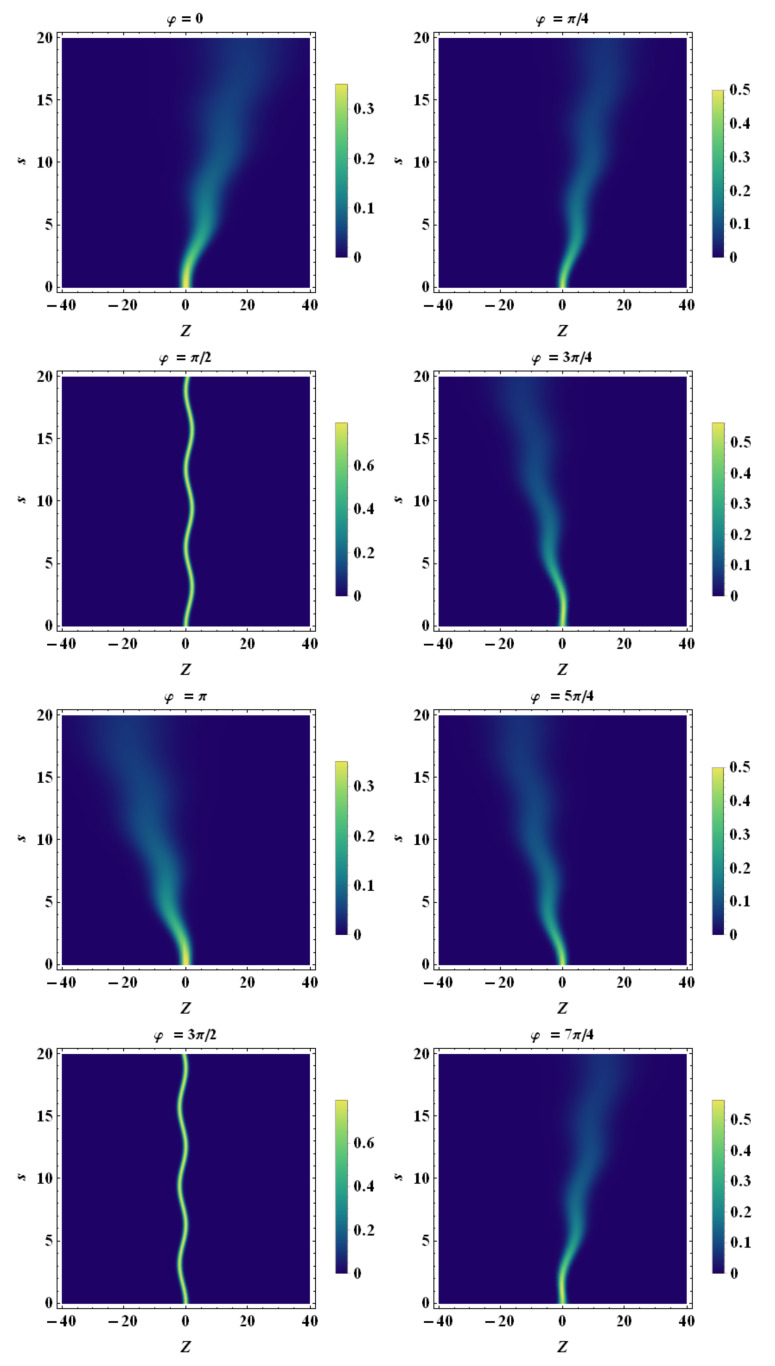
Density plot representation of tomogram as a function of *Z* and *s* for different angles φ. Here, we chose a non-constant acceleration with sinusoidal profile, i.e., $a\left(t\right)=a_{0}\sin\left(\omega s\right)$, with $a_{0}=1$ and ω=1. $\beta_{0}=0$, ${\dot{\beta }}_{0}=0$, $\sigma_{0}=1.0$, ${\dot{\sigma }}_{0}=0$, and $\lambda_{c}\equiv 1$. Note that the tomogram shapes exhibit periodic bending that is related to the temporal periodic character of the acceleration Similar to the other cases previously discussed, all of the tomograms exhibit a progressively increasing quantum spreading except the ones related to the angles φ=π/2 and φ=3π/2, which exhibit fixed spreading.

**Figure 11:**
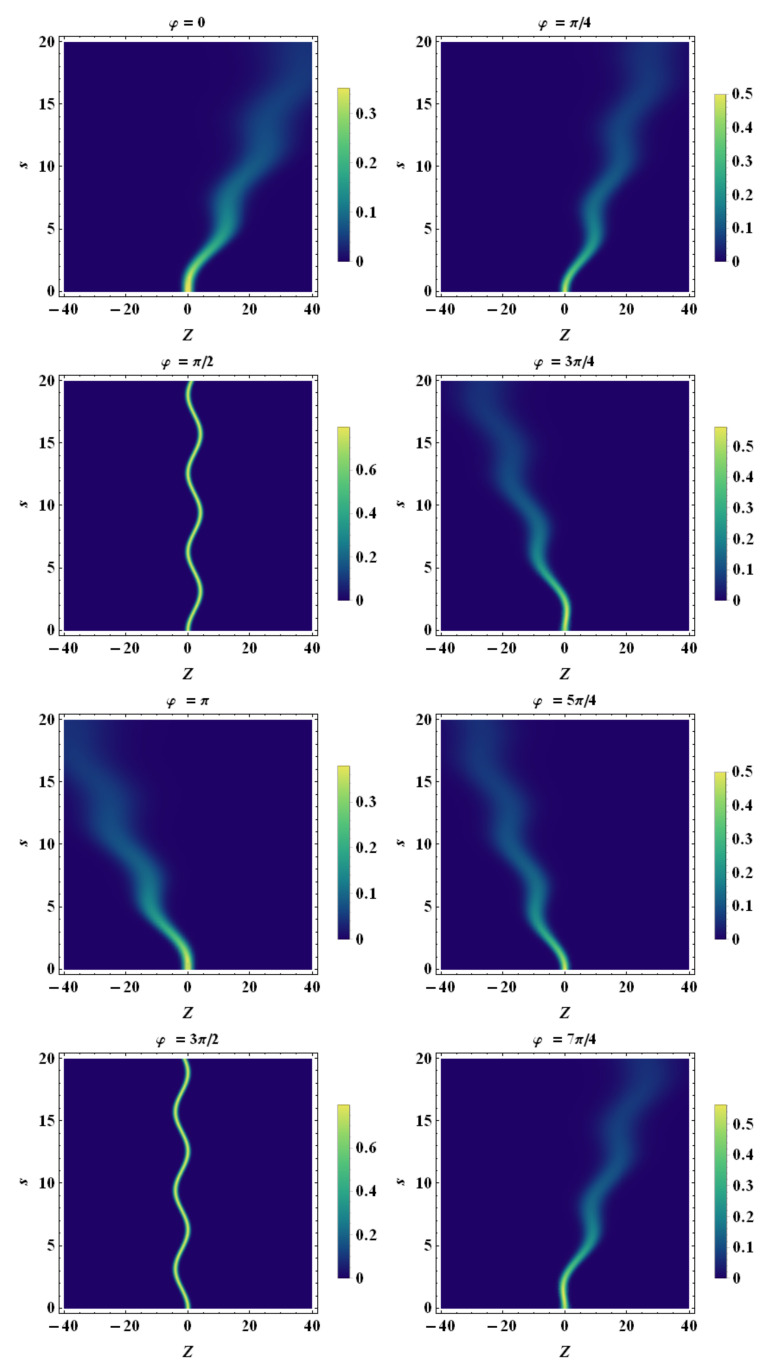
Density plot representation of tomogram as a function of *Z* and *s* for different angles φ. Here, we chose a non-constant acceleration with sinusoidal profile, i.e., $a\left(t\right)=a_{0}\sin\left(\omega s\right)$, with $a_{0}=2$ and ω=1. $\beta_{0}=0$, ${\dot{\beta }}_{0}=0$, $\sigma_{0}=1.0$, ${\dot{\sigma }}_{0}=0$, and $\lambda_{c}\equiv 1$. Note that the tomogram shapes exhibit periodic bending that is related to the temporal periodic character of the acceleration. Similar to the other cases previously discussed, all of the tomograms exhibit progressively increasing quantum spreading except the ones related to the angles φ=π/2 and φ=3π/2, which exhibit fixed spreading.

Let us consider the generic particle position *P*, at time *s*, along its trajectory in the phase space. Let us also denote by $\vec{OP}$ the position vector of the particle (with *O* being the origin of the phase-space reference frame). The particle motion can be projected along the direction of the tomographic *Z*-axis, which is inclined at the angle φ relative to the *z*-axis. The tomographic projection $Z_{p}$ can be expressed in terms of the tomographic angle φ and the current position of the particle in the phase space, employing the optical tomography transformation µ=cosφ, ν=sinφ and setting $Z_{p}=zµ+\lambda_{c}\nu p$, viz.

(60) $$Z_{p}=z_{p}\cos\varphi +\lambda_{c}{\dot{z}}_{p}\sin\varphi ,$$

yielding the tomographic map of the particle at position *P* (Figure 8, Figure 9, Figure 10 and Figure 11), Equation (41), in the form:

(61) $$w_{p}\left(Z,\varphi ,s\right)=\frac{1}{\sqrt{2\pi \sigma_{Z}^{2}\left(\varphi ,s\right)}}\exp\left\{-\frac{{\left(Z-Z_{p}\right)}^{2}}{2\;\sigma_{Z}^{2}\left(\varphi ,s\right)}\right\}\;.$$

In order to obtain a full tomographic representation of the wave packet at the instant *s*, we have, in principle, to scan all of the orientations of the tomographic axis with respect to the *z*-axis, i.e., we have to continuously change the tomographic angle φ. In this regard, two special cases are noteworthy: φ=0 and φ=π/2. The former is associated with the tomographic map that becomes the spatial distribution of the probability of the wave packet. Therefore, we have

(62) $$w\left(Z,r=1,\varphi =0,s\right)=\frac{1}{\sqrt{2\pi \sigma_{Z}\left(\varphi =0,s\right)}}\;\exp\left\{-\frac{{\left[Z-\beta (s)\right]}^{2}}{2\sigma_{Z}{\left(\varphi =0,s\right)}^{2}}\right\}\;,$$

where $\sigma_{Z}\left(\varphi =0,s\right)=\sigma \left(s\right)$ is the width of the wave packet at time *s*. For φ=π/2, the tomographic map becomes the distribution of the probability of the wave packet in the momentum space, namely:

(63) $$w\left(Z,r=1,\varphi =\pi /2,s\right)=\frac{1}{\sqrt{2\pi \sigma_{Z}\left(\varphi =\pi /2,s\right)}}\;\exp\left\{-\frac{{\left[Z-\beta (s)\right]}^{2}}{2\sigma_{Z}{\left(\varphi =\pi /2,s\right)}^{2}}\right\}\;,$$

where $\sigma_{Z}\left(\varphi =1/2,s\right)=\sigma_{p}\left(s\right)=\mathrm{const}.$ is the corresponding width.

## 7. Gaussian Tomographic Description for Non-Constant Acceleration

In this section, we consider the case of non-constant acceleration. In such a case, the spatiotemporal plots of the centroid motion in (z,s) configuration space and the related plots in both phase space (z,p) and tomographic space (Z,s) no longer have a parabolic shape because the non-constancy of the acceleration affects the bending of these plots. Below, in Section 7.1 and Section 7.2, we discuss two informative examples of Gaussian temporal burst and harmonic temporal oscillations of acceleration.

### 7.1. Gaussian Temporal Burst of Acceleration

As the first example, we consider a time-dependent acceleration with a Gaussian impulsive character (Gaussian temporal burst), i.e.,:

(64) $$a\left(s\right)=a_{0}\exp\left(-\frac{s^{2}}{\tau^{2}}\right)\;,$$

where $a_{0}$ is a constant. According to Section 3, in particular Equation (22), and Section 5, it is easy to determine the quantity β(s), i.e.,

(65) $$\beta_{0}-\frac{a_{0}\tau^{2}}{2}+{\dot{\beta }}_{0}s+\frac{1}{2}a_{0}\exp\left(-\frac{s^{2}}{\tau^{2}}\right)\tau^{2}+\frac{1}{2}\sqrt{\pi }a_{0}\tau sErf\left(\frac{s}{\tau }\right)\;,$$

and the quantity σ(s):

(66) $$\sigma \left(s\right)=\sigma_{0}\sqrt{1+\frac{2{\dot{\sigma }}_{0}s}{\sigma_{0}}+\left({\dot{\sigma }}_{0}^{2}+\lambda_{c}^{2}/4\sigma_{o}^{2}\right)\frac{s^{2}}{\sigma_{0}^{2}}}\;.$$

Then, the tomogram is straightforwardly obtained:

(67) $$w\left(Z,s,\varphi \right)=\frac{1}{2\pi \sigma_{Z}{\left(s,\varphi \right)}^{2}}\exp\left\{-\frac{{\left[Z-\left(\cos\varphi \beta \left(s\right)+\sin\varphi \dot{\beta }\left(t\right)\right)\right]}^{2}}{2\sigma_{Z}{\left(s,\varphi \right)}^{2}}\right\}\;,$$

where

(68) $$\sigma_{Z}\left(s,\varphi \right)=\sqrt{\frac{\lambda_{c}^{4}\sin\varphi^{2}}{4\sigma {\left(s\right)}^{2}}+{\left[\cos\varphi \;\sigma \left(s\right)\;+\;\lambda_{c}\;\sin\varphi \;\dot{\sigma }\left(s\right)\right]}^{2}}\;.$$

### 7.2. Sinusoidal Acceleration

The second example is given by the periodic time-variation of the acceleration of sinusoidal type:

(69) $$a\left(s\right)=a_{0}\sin\omega s$$

The function β(s) is determined in a way similar to that used in the preceding section, viz.

(70) $$\beta \left(s\right)=\beta_{0}+\left({\dot{\beta }}_{0}+\frac{a_{0}}{\omega }\right)s-\frac{a_{0}}{\omega^{2}}\sin\omega s,$$

while the expression for σ(s), $\sigma_{Z}\left(s,\varphi \right)$, and the tomogram, i.e., w(Z,s,φ), are formally identical to those in the previous case (see Equations (66) and (67), respectively). However, the different explicit time-dependencies of β make the tomograms in the two cases very different.

**Figure 12:**
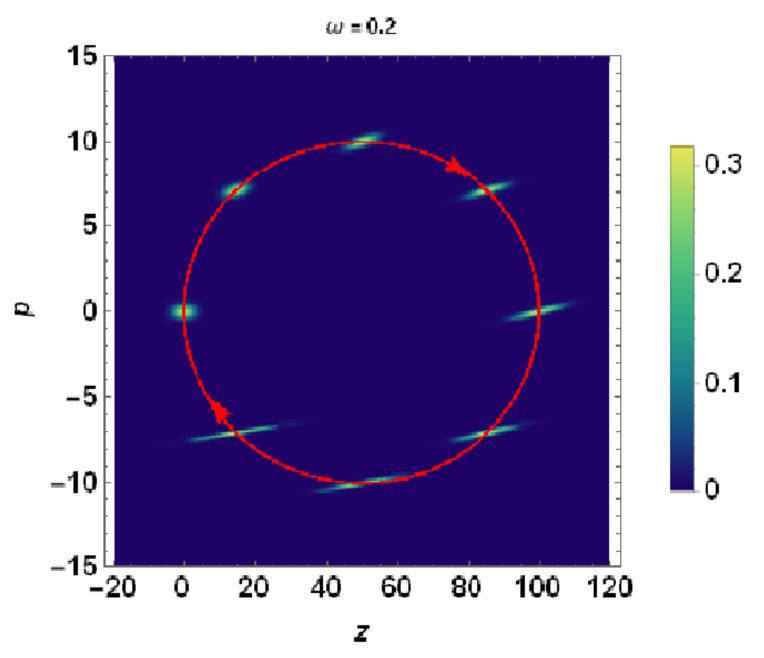
Density plot representation of the Wigner quasidistribution of the accelerating wavepacket experiencing a sinusoidal acceleration with parameters ω=0.2 and a=2. The Wigner quasidistribution is calculated at time interval Δs=0.125. The centroid of the distribution executes an elliptical trajectory in phase plane (see the red arrowed curve). The centroid of the Wigner quasidistribution at the initial time s=0 is located at z=p=0 of the phase plane. The modification underwent by Wigner quasidistribution with increasing time which results in significant spreading of the distribution in the configuration space (*z*-direction) is noticeable.

Before ending this section, it would be instructive to display for the readers how the quantum particle spot size (i.e., the density plot of the Wigner quasidistribution in phase space) looks similar to that in the presence of acceleration, as given by Equation (69). Figure 12 illustrates how both the particle spot size and the centroid move in phase space.

## 8. Conclusions and Remarks

In this paper, we reviewed different representations of a quantum wave packet in an accelerated frame. We used the model equivalent to the one of the quantum particle moving under the action of a time-dependent force. Examples of such motion are the ones of a charge in the time-varying uniform electric field or the motion of a mass in a uniform time-varying gravitational field.

The representations we used correspond to symplectic tomographic probability distributions identified with the quantum particle states as well as Fresnel and optical tomographic probability distributions related to the Wigner–Weyl phase-space representation by integral Radon transform.

We also discussed the Madelung representation of the pure state with the wave function considering the Gaussian form of wave functions for all of the representations under consideration. The explicit form of the Gaussian solutions of the Schrödinger equation rewritten in the tomographic form was obtained for a particular time dependence of the acceleration, namely, the case of constant acceleration, sinusoidal time dependence of acceleration, and the temporal burst form of acceleration.

The plots of quantum tomograms showing the centroid motion and details of packet extensions along the centroid motion were constructed for the representations of Gaussian quantum states used.

An important lesson demonstrated in this study is the fact that the quantum state can be identified with fair probability distributions containing complete information on the state, which is sufficient for obtaining all other functions identified with the state similar to, e.g., the Wigner function or the density matrix in the position representation.

New information available, in view of this probability representation of quantum states, is specific entropic-information relations for quantum tomograms characterizing the properties of quantum correlations in the system states, which are easy to write for probability distributions but which are not obvious in other representations, employing, e.g., the Wigner function. The study of this problem is already under way and it will be discussed in a future work.

Remarkably, the approach presented provides a very fruitful representation of both the quantum and quantum-like states in terms of a probability distribution with classical features, i.e., the marginal distribution. The latter differs substancially from the one provided by the Wigner quasidistribution, which is non-positive definite or from the quantum representation in terms of the probability amplitude (wave function for pure states, or density matrix, for mixed states). In particular, in quantum-like domain, within the context of EM radiation beams or charged-particle beams, both in paraxial approximation, the tomographic approach to beam transport and dynamics would be very helpful.

One of the principal aspects presented in this manuscript is the possibility to describe quantum phenomena in terms of a probability description with classical probability features, as provided by the tomogram. This way, the quantum states are not represented in terms of the wave function (i.e., the probability amplitude). Since the Radon transform is related to the wave function by means of a one-to-one mapping, the tomogram contains the same information provided by the wave function. This suggests another way to formulate quantum mechanics, based on a classical-like probability distribution, where the phase plays the role of hidden variable. Another important aspect is to provide a tomographic description in an accelerated system. We extensively presented examples to illustrate the tomograms embedded in a gravitational field that is originated in an accelerated frame.

Due to the richness of the material already presented in this manuscript, we decided not to include some other materials in progress concerning the entanglement studies and tomographic entropy. In a forthcoming investigation, we will dedicate a complete study to these subjects. 

## Data Availability

Not applicable.

## References

[1] Mancini S., Man’ko V.I., Tombesi P. (1995). Wigner Function and probability-distribution for shifted and squeezed quadratures. Quantum Semiclass. Opt..

[2] Mancini S., Man’ko V.I., Tombesi P. (1996). Symplectic tomography as classical approach to quantum systems. Phys. Lett..

[3] Mancini S., Man’ko V.I., Tombesi P. (1997). Classical-like description of quantum dynamics by means of symplectic tomography. Found. Phys..

[4] Vogel K., Risken H. (1989). Determination of quasiprobability distributions in terms of probability distributions for the rotated quadrature phase. Phys. Rev..

[5] Wigner E. (1932). On the Quantum Correction For Thermodynamic Equilibrium. Phys. Rev..

[6] Radon J. (1986). Über die Bestimmung von Funktionen durch ihre Integralwerte längs gewisser Mannigfaltigkeiten. Berichte über die Verhandlungen der Königlich-Sächsischen Akademie der Wissenschaften zu Leipzig, Mathematisch-Physische Klasse [Reports on the proceedings of the Royal Saxonian Academy of Sciences at Leipzig, mathematical and physical section], Leipzig: Teubner (69), 277 (1917); Translation: J. Radon, P.C. Parks(translator), On the determination of functions from their integral values along certain manifolds. IEEE Trans. Med Imaging.

[7] Glauber R.J. (1963). Coherent and incoherent states of the radiation field. Phys. Rev..

[8] Sudarshan E.C.G. (1963). Equivalence of semiclassical and quantum mechanical descriptions of statistical light beams. Phys. Rev. Lett..

[9] Husimi K. (1940). Some Formal Properties of the Density Matrix. Proc. Phys. Math. Soc. Jpn..

[10] Amosov G.G., Man’ko V.I., Orlov Y.N. (2009). Evolution equation of quantum tomograms for a driven oscillator in the case of the general linear quantization. Phys. Scr..

[11] Auletta G. (2000). Foundation and Interpretation of Quantum Mechanics.

[12] De Nicola S., Fedele R., Man’ko M.A., Man’ko V.I. (2005). Fresnel Tomography: A Novel Approach to Wave-Function Reconstruction Based on the Fresnel Representation of Tomograms. Theor. Math. Phys..

[13] De Nicola S., Fedele R., Man’ko M.A., Man’ko V.I. (2003). Tomography of solitons. J. Opt. B Quantum Semiclass. Opt..

[14] Leontovich M.A. (1944). On a method of solving the problem of propagation of electromagnetic waves near the surface of the earth. Izv. Akad. Nauk SSSR Ser. Fiz..

[15] Leontovich M.A., Fok V.A. (1946). About inconsistency of works by AA Vlasov on general theory of plasma and physics of solid body. Zh. Eksp. Teor. Fiz..

[16] De Nicola S., Fedele R., Man’ko M.A., Man’ko V.I. (2011). Fresnel entropic characterization of optical Laguerre-Gaussian beams. Phys. Lett..

[17] Fedele R., Miele G. (1991). A thermal-wave model for relativistic charged-particle beams. Il Nuovo C. D.

[18] Fedele R., Shukla P.K. (1992). Self-consistent interaction between the plasma wakefield and the driving relativistic electron beam. Phys. Rev..

[19] Fedele R., Miele G., Palumbo L., Vaccaro V.G. (1993). Thermal Wave Model for Nonlinear Longitudinal Dynamics in Particle Accelerators. Phys. Lett..

[20] Anderson D., Fedele R., Vaccaro V.G., Lisak M., Berntson A., Johansson S. (1999). Modulational instabilities within the thermal wave model description of high-energy charged particle beam dynamics. Phys. Lett. A.

[21] De Nicola S., Fedele R., Man’ko V.I. (1995). Coherent states for particle beams in the thermal wave model. Phys. Scr..

[22] Fedele R., Man’ko V.I. Quantum-like corrections and tomography in beam physics. Proceedings of the European Particle Accelerator Conference EPAC98 (1998).

[23] Fedele R., Man’ko V.I. (1998). Quantumlike Corrections and Semiclassical Description of Charged-Particle Beam Transport. Phys. Rev..

[24] De Nicola S., Fedele R., Man’ko M.A., Man’ko V.I. (2009). Entropic uncertainty relations for electromagnetic beams. Phys. Scr..

[25] De Nicola S., Fedele R., Man’ko M.A., Man’ko V.I. (2007). New inequalities for tomograms in the probability representation of quantum states. Theor. Math. Phys..

[26] Von Neumann J. (1932). Mathematische Grundlagen der Quantenmechanik.

[27] Ermakov V.P. (1880). Second-order differential equations, Conditions of complete integrability. Univ. Izv. (Kiev).

[28] Pinney E. (1950). The nonlinear differential equation *y*^″^ + *p*(*x*)*y* + *c**y*^−3^ = 0. Proc. Am. Math. Soc..

[29] Fiore G., Gouba L. (2011). Class of invariants for the two-dimensional time-dependent Landau problem and harmonic oscillator in a magnetic field. J. Math. Phys..

[30] Landau L.D., Lifsits E.M. (1957). Mechanics.

[31] Man’ko V.I., Mendes R.V. (1999). Non-commutative time-frequency tomography. Phys. Lett. A.

